# Osteoprotegerin, Pericytes and Bone-Like Vascular Calcification Are Associated with Carotid Plaque Stability

**DOI:** 10.1371/journal.pone.0107642

**Published:** 2014-09-26

**Authors:** Jean-Michel Davaine, Thibaut Quillard, Régis Brion, Olivier Lapérine, Béatrice Guyomarch, Thierry Merlini, Mathias Chatelais, Florian Guilbaud, Meadhbh Áine Brennan, Céline Charrier, Dominique Heymann, Yann Gouëffic, Marie-Françoise Heymann

**Affiliations:** 1 INSERM, UMR 957, Nantes, France; 2 Université de Nantes, Nantes atlantique universités, Nantes, France; 3 Centre Hospitalier Universitaire, Nantes, France; 4 Institut du Thorax, Nantes, France; Brigham and Women’s Hospital, Harvard Medical School, United States of America

## Abstract

**Background and Purpose:**

Vascular calcification, recapitulating bone formation, has a profound impact on plaque stability. The aim of the present study was to determine the influence of bone-like vascular calcification (named osteoid metaplasia = OM) and of osteoprotegerin on plaque stability.

**Methods:**

Tissue from carotid endarterectomies were analysed for the presence of calcification and signs of vulnerability according to AHA grading system. Osteoprotegerin (OPG), pericytes and endothelial cells were sought using immuno-histochemistry. Symptoms and preoperative imaging findings (CT-scan, MRI and Doppler-scan) were analyzed. Human pericytes were cultured to evaluate their ability to secrete OPG and to influence mineralization in the plaque.

**Results:**

Seventy-three carotid plaques (49 asymptomatic and 24 symptomatic) were harvested. A significantly higher presence of OM (18.4% vs 0%, p<0.01), OPG (10.2% of ROI vs 3.4% of ROI, p<0.05) and pericytes (19% of ROI vs 3.8% of ROI, p<0.05) were noted in asymptomatic compared to symptomatic plaques. Consistently, circulating OPG levels were higher in the plasma of asymptomatic patients (3.2 ng/mL vs 2.5 ng/mL, p = 0.05). *In vitro,* human vascular pericytes secreted considerable amounts of OPG and underwent osteoblastic differentiation. Pericytes also inhibited the osteoclastic differentiation of CD14+ cells through their secretion of OPG.

**Conclusions:**

OPG (intraplaque an plasmatic) and OM are associated with carotid plaque stability. Pericytes may be involved in the secretion of intraplaque OPG and in the formation of OM.

## Introduction

Arterial calcification (AC) is independently associated with increased cardiovascular morbidity and mortality [1], and its development in atheromatous lesions impacts deeply on plaque stability [2]. AC is now reconized as a highly-regulated process which recapitulates bone tissue formation and homeostasis [3]–[5]. Observation of bone-tissue, named osteoid metaplasia (OM) and even of bone marrow in vessels has been reported many years ago [6]. Osteoblast-like cells have been identified in atheromatous lesions and while the exact nature of these cells remains to be fully determined, several lines of evidence support that pericytes are serious candidates [7]–[9]. Also, giant multinucleated cells positive for tartrate-resistant acid phosphatase (TRAP) have been identified in atherosclerosis lesions [10]. They most likely originate from intraplaque macrophages, which have the ability to differentiate into osteoclast-like cells in the presence of RANK-Ligand [11]. Comprehension of the mechanisms underlying the formation of mineralized tissue within carotid atherosclerosis lesions is of high importance since calcification greatly influences the plaque stability and the consecutive risk of stroke [2], [3], [12]. At the molecular level, the OPG- RANK-RANKL triad, fundamental in bone homeostasis is also present in atheroma lesions [13], further suggesting that atheromatous calcification micro-environment reproduce landmark characteristics of bone tissue. Osteoprotegerin has been positively associated with the severity of coronary artery disease and cardiovascular mortality in humans [14]–[16], but also with the severity of peripheral artery disease [17], symptomatic carotid lesions [18], and vulnerable carotid plaques [19]. However, its exact clinical relevance regarding the development of OM and plaque stability in carotid lesions remains to be elucidated. Importantly, if well-defined characteristics of vulnerable carotid plaques are available (intraplaque hemorrage, thin fibrous cap, large necrotic core) [20],their use in the context of preoperative patient risk-assessment is limited and clinically relevant markers of carotid plaque vulnerability are awaited with much anticipation.

The aim of the present study was to investigate the influence of OM on carotid plaque stability and to test the hypothesis that pericytes and OPG are determinant in this process.

## Materials and Methods

### Patients, biological samples and imaging data

From February 2008 to June 2010, atheromatous plaques were harvested from 73 patients undergoing carotid endarterectomy in our center. Patients presenting with non-atherosclerotic peripheral arterial disease, thrombosis and/or restenosis were excluded. Detailed demographic and clinical characteristics recorded were: age, sex, treatments, cardiovascular risk factors, medical history, and serum biochemistry analyses. All participating patients to the study gave a written informed consent. The clinical research protocol was approved by our institutional medical ethics committee (Nantes University Hospital Ethics Committee). Doppler-ultrasound (Doppler-us) examination associated with either angio-MRI or angio CT-scan were carried out to assess the characteristics of the lesions and intra-cerebral collaterality, as well as potential cerebral lesions. The results of these examinations were thoroughly reviewed by one of the author who was blinded to the symptomatic or asymptomatic status of corresponding patient. Based on the imaging findings, the calcic burden of the plaques was determined and plaques were categorized as highly calcified, moderately calcified, mildly calcified or non-calcified. Carotid plaques were considered as symptomatic when carotid stenosis ≥50–70% was associated with the onset of ipsilateral transient ischemic attack or stroke or retinal ischemia and in the absence of other cause following extensive pre-operative workup. In this case, surgery associated with best medical treatment (antiplatelet agent, statin therapy and correction of cardiovascular risk factors) was indicated [21], [22]. Surgery consisted of endarterectomy and was performed within 2–4 weeks of the ischaemic event [23], [24]. Indication for carotid endarterectomy for asymptomatic carotid lesions was stenosis ≥60–70% according to NASCET criteria and with acceptable life expectancy [22]–[25].

Blood samples were collected in EDTA tubes 24 h prior to surgery, and plasma was isolated by centrifugation, aliquoted and stored at −80°C until use.

### OPG measurement

OPG levels were measured in plasma and cell culture supernatants by a validated enzyme immunoassay using commercially available matched antibodies (DY805 ELISA kit, R&D Systems, Minneapolis, Minn., USA). Osteoblastic pericyte-conditioned medium was obtained as follow: pericytes were cultured for 16 to 18 days in an osteoblastic-inducing medium. Then, cells were washed and a new medium, containing only 1% FCS, was used. After 48 hours the supernatant was harvested, centrifuged, aliquoted and stored at −80°C. Experiments were repeated three times in duplicate.

### Histological and immunological analyses

Carotid plaques were removed by endarterectomy at the bifurcation from within the lumen as a single specimen. The atherosclerotic plaques were immersed in physiological serum, transferred to ice, and fixed in 10% formalin overnight, decalcified in Sakura TDE 30 fluid for 24 h, and embedded in paraffin. Adjacent sections (5 µm thick, 20 sections per patient) were stained with (i) hematoxylin eosin (HE), (ii) masson trichrome, or incubated with monoclonal antibodies directed against (iii) CD31 (dilution 1/20, M823, Dako), (iv) NG2 (frozen sections, dilution 1/25, MAB2585, R&D Systems, Minneapolis, MN), (v) CD146 (dilution 1/200, AB75769, Abcam, Paris, France), (vi) smooth muscle actin (dilution 1/75, MAB1420, R&D Systems), and (vii) osteoprotegerin (dilution 1/25, AF805, R&D Systems). Plaques were analyzed for the presence of calcification which were categorized in sheetlike calcifications, nodular calcifications, clear center calcifications and osteoid metaplasia, as previously described [26]. The latter type consisted in typical mature bone with lamellar structure and in some cases bone-marrow. The sections were also examined separately by two authors, who were blinded to the status of the plaque, for the presence of signs of vulnerability according to Stary’s classification [20]: plaques with thick fibrous or fibrocalcic content covering central necrotic core were considered as stable (types IV, Vb and c) as opposed to unstable plaques that presented with thin or ruptured fibrous cap, large necrotic core (type IV and Va) or hemorrhage or ulceration (type VI).

Imaging of the sections was obtained with the NanoZoomer device (Hamamatsu Photonics, Hamamatsu, Japan) and staining quantification was carried out using Image J software (Image J, NIH, Bethesda, MD). Results were expressed as a percentage of region of interest (ROI).

### Cell culture and differentiation assays

Reagents were purchased from Sigma (St Louis, MO), unless stated otherwise. Human placenta-derived pericytes (C12980) were grown in pericyte growth medium (C28040) (PromoCell, Heidelberg, Germany). The pericytes were subsequently placed for 18 days in an osteogenic culture medium containing vitamin D3 (10^−8 ^M), dexamethasone (10^−7 ^M), ascorbic acid (50 µg/ml) and β-glycerophosphate (10 mM).

Human bone marrow-derived mesenchymal stem cells (MSC) from healthy donors [27] were grown in Dulbecco’s Modified Eagle Medium (DMEM) supplemented with 10% fetal calf serum, 1 ng/mL basic Fibroblast Growth Factor (bFGF; R&D systems), 100 U/ml penicillin, 100 U/ml streptomycin and 2 mM L-glutamine. Adherent cells were stored frozen at passage two after characterization by flow cytometry (CD45^−^, CD34^−^, CD105^+^, CD73^+^ and CD90^+^, purity≥99%). MSC were plated at a density of 10^4^ cells/cm^2^ in 96-well plates. On day 3 and until day 28, osteoblastic differentiation was realized and assessed using alizarin red staining [28].

CD14^+^ peripheral blood mononuclear cells (PBMC) were isolated from healthy donors using Ficoll gradient and CD14 microbeads with MACS separators (Miltenyi Biotec, Bergisch Gladbach, Germany), checked by flow cytometry (CD14^+^, CD3^−^, purity≥95%), and stored frozen in liquid N_2_ until use. CD14^+^ cells were grown in 96-well plates (35000 cells/well) using alpha-MEM (Lonza, Verviers, Belgium) supplemented with 10% FCS and 25 ng/mL of recombinant macrophage colony stimulating factor (MCSF) (Sigma, St Quentin Fallavier, France). Starting on day 3 and until day 14, the medium was supplemented with RANK-L (100 ng/mL), with or without 50–100 ng/mL of either recombinant TNFα or IL-6 (R&D Systems) and with 20% of pericyte-conditioned medium or control medium [28]. On day 14, multinucleated cells were stained for tartrate-resistant acid phosphatase (TRAP) using the Leukocyte Acid Phosphatase Assay kit, and images were analyzed on a D70 camera microscope (Olympus, Hamburg, Germany).[29] Neutralizing antibody directed against human OPG (TNFRSF11B antibody, R&D Systems) was used at a concentration of 0.7 µg/mL, as required by the instructions provided.

### Gene expression

Total RNA was extracted from the pericytes in a subset of wells using TriZol (Life Technologies, Courtaboeuf, France) and reverse transcribed. The gene expression levels for osteoblastic markers, Runt-related transcription factor 2 (RunX2) (F-gtgcctaggcgcatttca/R-ggctcttcttactgagagtggaag), osteocalcin (F-ggcgctacctgtatcaatgg/R-tcagccaactcgtcacagtc) and alkaline phosphatase (ALP) (F-aacaccacccaggggaac/R-ggtcacaatgcccacagatt) were assessed with real time PCR using SYBR green (Bio-Rad, Marnes la Coquette, France).

### Statistics

Throughout the manuscript, the data are presented as a mean ± SEM, unless stated otherwise. Statistical analyses were performed using SSPS 10.0 software. The data were compared either using the Chi-square test, or the two-sample Student’s t-test, or a non parametric Mann and Whitney test when appropriate. A p<0.05 was considered statistically significant.

## Results

### Osteoid Metaplasia is a specific feature of asymptomatic carotid plaques

**Figure 1:**
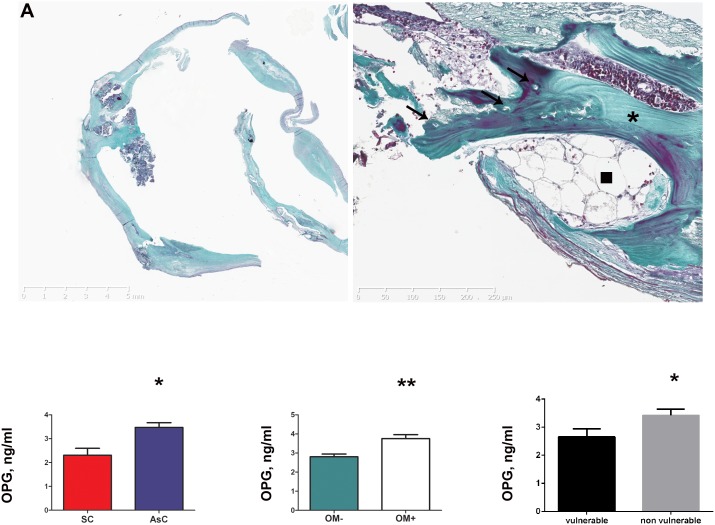
(A) Representative sections of asymptomatic carotid plaque (left panel) with higher magnification showing dense lamelar structure (*), osteocytes (arrow), and even bone marrow (arrowhead) typical of osteoid metaplasia (right panel), Masson’s Trichrome staining. (B) Patients presenting with asymptomatic plaques (AsC) had significantly higher OPG plasmatic levels than symptomatic patients (SC) (left panel). Similar result was observed when comparing OM+ and OM– groups (middle panel), and histologically-assessed vulnerable and non-vulnerable plaques (right panel), using Elisa assays.

Out of the 73 carotid plaques, OM ( = bone-tissue) was noted in 18.4% (9/49) of the asymptomatic and in none (0/24, 0%) of the symptomatic carotid lesions (p = 0.02) (Fig. 1A and Table 1). Analysis of imaging data showed that 62.5% of asymptomatic carotid plaques were highly or moderately calcified vs 8.4% of the symptomatic lesions (p<0.001) (Table 2).

**Table 1:** Comparison between asymptomatic and symptomatic carotid groups showed completely diffferent calcic, OPG and CD146+ cells patterns.

Carotids	SC	AsC	p*-value*
**OM+, n (%)**	0 (0%)	9 (18%)	**0.02**
**OM−, n (%)**	24 (100%)	40 (82%)	
**Circulating OPG (ng/ml)**			**0.05**
**n**	24	49	
**mean±SD**	2.5±1.6	3.2±1.5	
**Intraplaque OPG (% ROI)**			**<0.001**
**n**	23	39	
**mean±SD**	3.4±4.5	10.3±6.6	
**Intraplaque CD146 (% ROI)**			**<0.001**
**n**	23	47	
**mean±SD**	2.3±2.8	6.4±5.0	
**Intraplaque CD31(% ROI)**			0.230
**n**	23	46	
**mean±SD**	1.2±1.4	2.3±4.1	

OM+: presence of osteoid metaplasia, OM−: absence of osteoid metaplasia.

**Table 2:** Characteristics of the lesions, clinical symptoms and imaging modalities.

	SC (24/24)	AsC (n = 32/49)	p*-*value
**Degree of stenosis**			**0.04**
60–69, n(%)	2 (8.3%)	0 (0%)	
70–79, n(%)	8 (33.3%)	20 (62.5%)	
>80, n(%)	14 (58.3%)	12 (37.5%)	
**TIA, n(%)**	8 (33.3%)	-	-
**Stroke, n(%)**	15 (62.5%)	-	-
**Preoperative imaging modalities**			
Doppler US, n(%)	24 (100%)	32 (100%)	-
MRI, n(%)	13 (54.2%)	26 (81.3%)	0.06
CT-scan, n(%)	23(95.8%)	8 (25%)	**<0.001**
**Calcification**			**0.001**
Highly calcified, n(%)	1 (4.2%)	1 (3.1%)	
Moderate, n(%)	1 (4.2%)	19 (59.4%)	
Mild or none, n(%)	8 (37.4%)	6 (18.8%)	
NA, n(%)	13 (54.2%)	7 (18.7%)	

Degree of stenosis are reported according to NASCET criteria. Symptomatology is reported as transient ischemic attack (TIA) or stroke. Previous to surgery patients underwent Doppler-ultrasound (Doppler-us) in all cases and another imaging modality that was whether Magnetic Resonance Imaging (MRI) or Computerized Tomography Scanner (CT-scan).

### Osteoid Metaplasia is more frequent in histologically-assessed stable carotid plaques

Osteoid metaplasia was only observed in asymptomatic lesions. Vulnerability status of the plaques was determined based on the modified AHA classification [20]. Symptomatic lesions had histological signs of vulnerability in 91.7% (22/24) of the cases. Asymptomatic lesions had no histological sign of vulnerability in 88.1% (37/42) of the cases. Consistently, OM was observed in only 7.4% (2/27) of the vulnerable plaques and in 17.9% (7/39) of the non-vulnerable plaques (p = 0.220).

### Circulating OPG levels correlate positively with the asymptomatic nature of carotid plaques, the presence of OM and histologically-assessed plaque stability

The demographic and cardiovascular characteristics of the patients were compared. (Table S1 in File S1). Patients with asymptomatic carotid plaques had higher levels of LDL cholesterol (0.9±0.3 g/L vs 0.7±0.3 g/L, p = 0.02) and presented more frequently with associated peripheral arterial disease (PAD) (39% vs 19%, p = 0.05). Patients with symptomatic carotids were more frequently under 2 antiplatelet agent (50% vs 27%, p = 0.05). Levels of circulating OPG were found to be significantly higher in the plasma of patients presenting with asymptomatic carotid lesions compared to symptomatic carotid lesions (3.2±1.5 ng/mL vs 2.5±1.6 ng/mL, p = 0.05) (Fig. 1B and Table 1). Likewise, patients with OM in their plaques had significantly more OPG in their plasma compared to patients without (3.5±1.2 ng/mL vs 2.3±1.3 ng/mL, p = 0.03) (Fig. 1B). Finally and consistently, OPG levels were higher in the plasma of patients presenting with stable plaques compared to patients presenting with histological signs of plaque vulnerability (3.42±0.22 ng/mL vs 2.66±0.28 ng/mL, p = 0.03) (Fig. 1B).

### Cellular OPG expression is higher in asymptomatic carotid plaques

**Figure 2:**
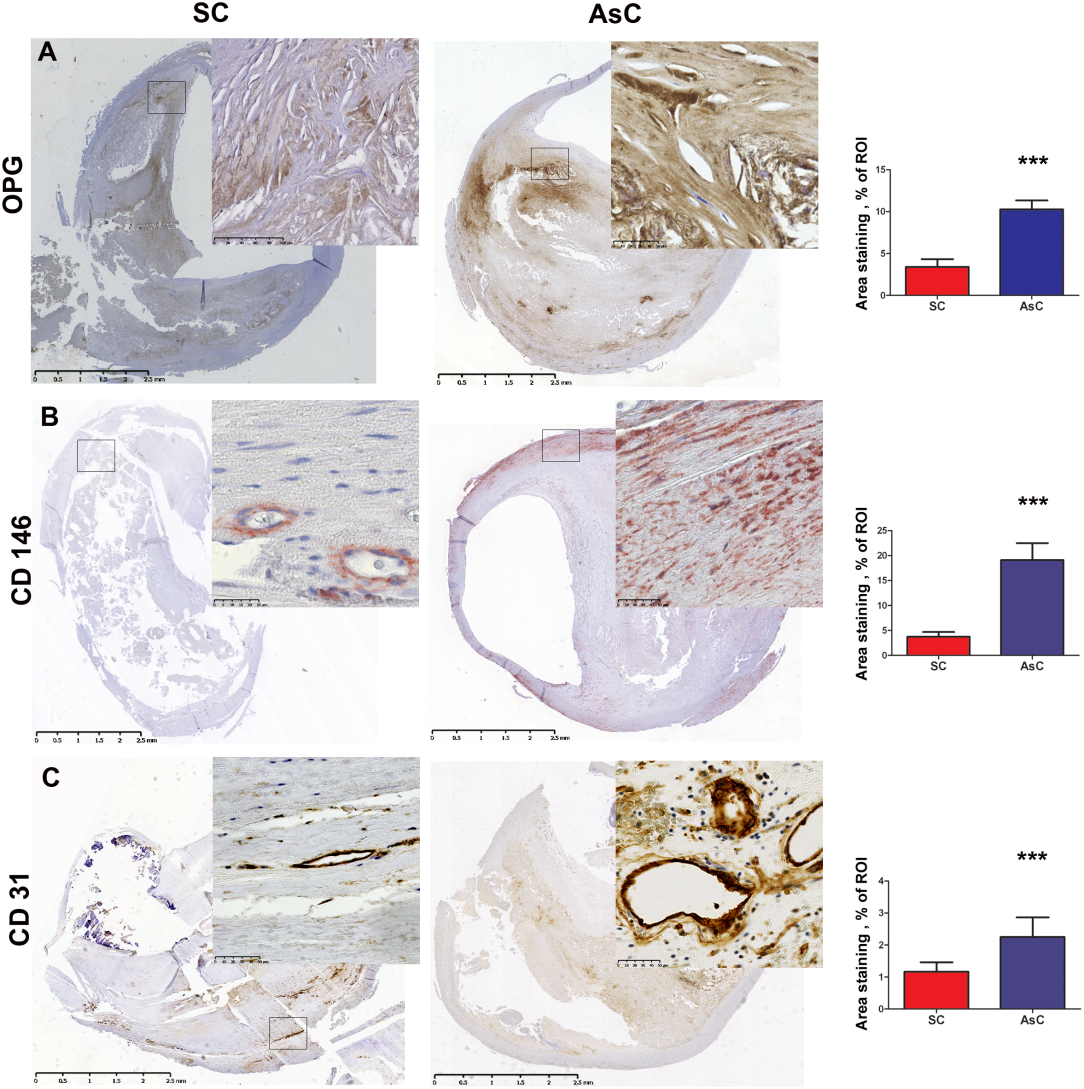
Immunohistochemistry experiments were used to compare symptomatic carotid plaques (SC) and asymptomatic carotid plaques (AsC). Asymptomatic lesions presented higher OPG infltration, p<0.001 (A), higher CD146^+^ pericyte infiltration, p<0.001 (B), and higher CD31^+^ endothelial cell infiltration, p = 0.23 (C). Representative images are on the left with corresponding quantification on the right.

In line with the plasmatic findings, asymptomatic carotid lesions contained significantly more OPG than symptomatic carotid lesions (10.3±6.6 vs 3.4±4.5, % area staining, ROI p<0.001) (Fig. 2A and Table 1).

### Intense vascular pericyte presence accompanies calcified asymptomatic carotid lesions

**Figure 3:**
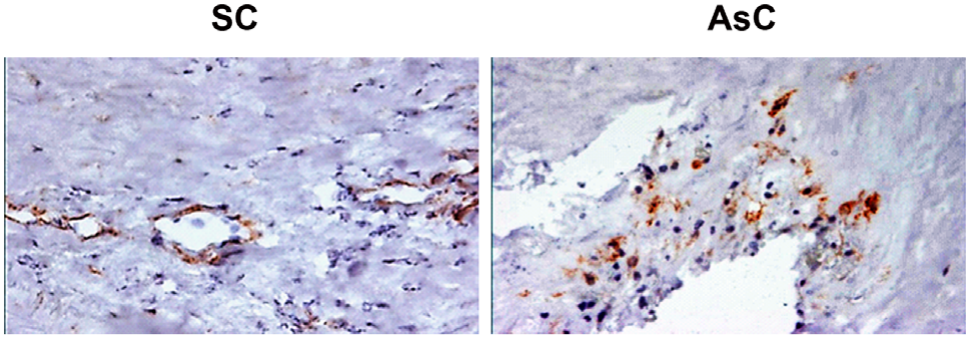
NG2 staining was performed on a subset of lesions. The findings were similar to those obtained with CD146: NG2 pericytes were found in a higher proportion in the asymptomatic (AsC) carotid lesions (right panel) than in the symptomatic (SC) carotid lesions (left panel).

As we hypothesized that pericytes could be involved in the onset of mineralized structure in these plaques and in the secretion of OPG, pericyte presence in the plaques was assessed. Importance of CD146^+^ pericyte staining was significantly higher in the asymptomatic carotid lesions compared to the symptomatic carotid lesions (6.4±5.0 vs 2.3±2.8, % area staining, ROI, p<0.001) (Fig. 2B and Table 1). A subset of sections were stained for NG2 pericyte marker, confirming the results obtained with CD146 (Fig. 3).

A difference towards more CD31^+^ endothelial staining in asymptomatic lesions was noted but without reaching statistical significance (2.3±4.1 vs 1.2±1.4% area staining, ROI, p = 0.230 (Fig. 3C and Table 1). Finally, analysis of smooth muscle actin staining showed no significant difference between the asymptomatic and symptomatic carotid lesions (Figure S1 in File S1).

### Human pericytes are able to differentiate into osteoblast-like cells and to mineralize *in vitro*

**Figure 4:**
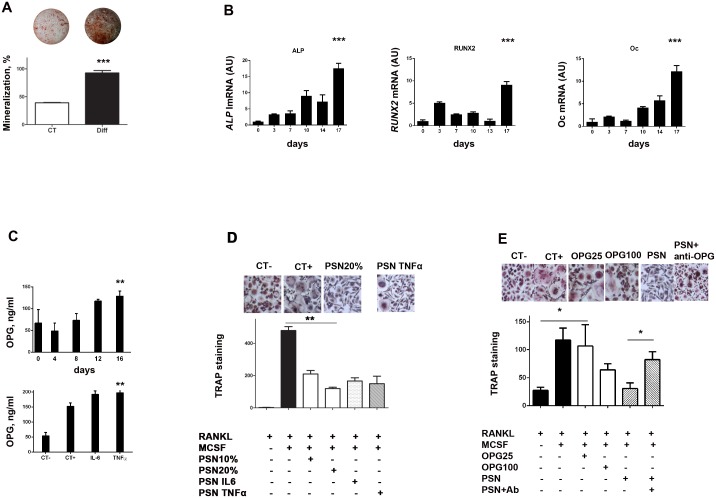
(A) Human primary pericytes underwent osteoblastic differentiation when cultured in an osteogenic media, as shown by Alizarin red staining. Representative images are above corresponding bars. (B) PCR experiments confirmed that during this process, pericytes increasingly expressed the ALP (p<0.0001), Runx2 (p<0.0001) and osteocalcin (Oc) (p<0.0001) characteristic markers of osteoblasts. (C) CD14+ cells were cultured during 14 days with standard media (CT−) or with addition of M-CSF and RANKL (CT+), after which TRAP staining was realized and giant multinucleated cells counted. Addition of pericytes supernatant (PSN) to the medium (in the proportion of 10% and 20%) dose-dependently inhibited the osteoclastic differentiation. A similar result was obtained when IL-6 and TNF-α pericyte-conditioned supernatant were used. Representative images are displayed above the bars. (D) Elisa experiments showed that pericytes expressed increasing amounts of OPG along their osteoblastic differentiation (upper graph) but also under inflammatory stimulation by IL-6 and TNF-α (lower graph). (E) CD14+ cells were cultured to undergo osteoclastic differentiation as in (C). Addition of OPG (25 ng/ml and 100 ng/ml), inhibited the formation of multinucleated cells. Addtion of PSN produced similar effect. Finally, addition of an antibody directed against OPG (PSN + anti-OPG) significantly reversed this inhibition. All experiments were realized three times in triplicate. CT-: media with RANKL, CT+: media with RANKL and MCSF.

To further investigate if pericytes could be responsible for the intense mineralization and OPG secretion observed in asymptomatic carotid lesions, human primary pericytes (CD31^−^, CD146^+^ and NG2^+^, Figure S2 in File S1) were studied *in vitro*. Primary pericytes were able to mineralize when grown in osteogenic media (Fig. 4A). Simultaneously, PCR analysis showed that the expression of the osteoblastic differentiation markers alkaline phosphatase, RunX2, and osteocalcin were significantly increased (ALP: 17.6-fold, p<0.0001, RunX2: 9.1-fold, p<0.0001, osteoclacin: 12.2-fold, p<0.0001) (Fig. 4B). At baseline, human pericytes secreted elevated amount of OPG in comparison to smooth muscle cells and endothelial cells (Figure S3 in File S1). Pericyte-derived OPG secretion was significantly increased during their osteoblastic differentiation as well as under inflammatory stimulation (Fig. 4C).

### Pericytes inhibit the RANKL-dependent differentiation of pre-osteoclasts into mature osteoclasts through expression of OPG

Macrophages, abundantly present in the atherosclerotic plaques represent a potential source of osteoclast-like cells in atheromatous calcified plaques. To understand the influence of pericytes on the mineralization balance within the plaque, we tested the effect of pericyte-conditioned medium on osteoclastogenesis. Pericytes were cultured under osteoblastic conditions. After 16 to 18 days, we added fresh FCS-deprived culture medium for an additional 48 hours before collecting the supernatant. We observed that this osteoblastic pericyte-conditionned medium inhibited the RANKL- dependent differentiation of PBMC into osteoclasts, as shown by the significant reduction of TRAP-positive multinucleated cells *in vitro* (Fig. 4D). This phenomenon was still observed when inflammatory-conditionned pericyte supernatants (TNFα or IL6- pericyte-conditionned medium) were used (Fig. 4D). Since pericytes secreted increasing amounts of OPG when cultured in osteogenic medium as well as under TNFα and IL-6 stimulation (Fig. 4C), we tested the effect of a neutralizing monoclonal antibody directed against OPG and observed a significant reversion of this inhibiting effect (Fig. 4E).

## Discussion

### OPG is a promising predicitive marker for carotid plaque stability

The main finding of this study is the significant difference in terms of OPG profile, both plasmatic and intraplaque, between asymptomatic and symptomatic carotid plaques. Both OPG presence in the plaque and circulating OPG levels were found to be significantly higher in the asymptomatic compared to the symptomatic carotid lesions. OPG is an ubiquitous molecule, secreted by cells of the cardiovascular system (endothelial cells, smooth muscle cells, pericytes) but also by other tissues such as bone and the immune sytem [11], [30], [31]. The circulating levels of OPG thus result from the activity of these various organs. However, here we observed that circulating OPG levels strictly mirrored intraplaque OPG presence and correlated positively with carotid plaque stability, suggesting that OPG plasmatic levels reflect the vulnerability of the carotid lesions. In contrast, imaging modalities could not accurately distinguish the symptomatic/vulnerable plaques from the asymptomatic/more stable plaques, confirming that no robust imaging markers for plaque vulnerability are currently available. As a result, plasmatic OPG should be seriously considered as an interesting potential marker for carotid plaque stability.

### Osteoid Metaplasia is a critical feature of asymptomatic carotid lesions

The calcic composition of the plaque has a considerable impact on its clinical behavior (thrombosis, dissection, embolism) or on its clinical response to treatment (stent fracture, in-stent restenosis). A significant difference in terms of vascular calcification was observed between asymptomatic and symptomatic carotid plaques. Histological analyses and imaging findings showed that asymptomatic carotid lesions were significantly more calcified than symptomatic carotid lesions. Interestingly, the main difference in terms of calcic pattern concerned OM, a highly-evolved bone-like type of vascular calcification, since almost 20% of the asymptomatic (versus none of the symptomatic) displayed OM. In a previous work, Hunt et al. made similar observation, showing that highy-calcified carotid lesions were less prone to induce stroke [3]. Notably, they found a similar (13%) rate of bone formation in the most calcified lesions. Recent works have established the importance of microcalcifications in plaque stability [32], [33], strongly suggesting that the presence of microcalcifications in the thin fibrous cap covering atherosclerotic lesions increase the risk of rupture. This is consistent with the present results: microcalcification may destabilize the lesions by introducing a mismatch at the interface between soft and hard calcified tissue, while highly-evolved large calcification could ultimately stabilize the lesion. Consequently, OM should not only be considered as a marker for highly-evolved lesions, but also as a qualitative and informative marker for plaque vulnerability.

### Development of OM in the plaques: the more OPG, the more bone?

The exact influence of OPG remains subject to controversy. The phenotype of OPG−/− mice suggests a protective role against vascular calcification development [34]. In contrast, OPG levels in humans correlate positively with the calcic burden of the plaque and the severity of the cardiovascular disease [15], [16]. In bone tissue, OPG is secreted by osteoblasts and acts as a soluble decoy receptor for RANK-Ligand. As a result, osteoclast differentiation and activation and finally bone resorption are inhibited. Here, we consistently observed that asymptomatic carotid lesions, displaying OM, had the highest intraplaque OPG level. This high mineral presence within the plaque may ultimately result in a more stable phenotype. Taken together, these results suggest that an intense secretion of OPG in carotid plaques promotes the development of OM, which in turn stabilizes the lesion, leading to the asymptomatic phenotype.

Previous works have shown different results [18], [35]. Straface et al. suggested that high serum OPG levels might be associated with plaque instability, however, if the plaques were histologically-assessed regarding their vulnerability status, no information on the clinical symptoms of the patients were provided, limiting the clinical relevance of their conclusions. Similarly, Golledge et al. also observed elevated OPG levels in symptomatic carotid lesions, but no histological data regarding plaque vulnerability were provided. In contrast, we here provide both clinical and histological data of the carotid lesions as well as detailed imaging data analysis. The results consistently and strongly indicate that higher OPG plasma levels are to be found in patients with asymptomatic carotid lesions.

### Implication of pericytes in the differential regulation of bone-like vascular calcification

It is now established that bone formation and arterial calcification share many similarities. In bone, OPG is secreted by osteoblasts and acts as a decoy receptor for RANKL. Osteoprotegerin prevents RANKL fixation on its receptor RANK at the surface of osteoclasts, resulting in the inhibition of osteoclast proliferation and differentiation. This process finely tunes bone formation and resorption [36]. In the arterial wall, osteoblast precursors and the OPG/RANKL/RANK axis have clearly been identified [13]. During arterial calcification formation, cells with osteochondrogenic potential differentiate along the osteoblastic lineage, yet the exact nature of these cells (smooth muscle cells, endothelial cell, calcifying vascular cells, pericytes) remains to be elucidated [37]. Vascular smooth muscle cells represent the most common cell type studied. They have been shown to have important phenotypic plasticity and when exposed to hydroxyapatite cristals, high concentrations of phosphate or inflammatory cytokines, vascular smooth muscle cells differentiate into osteoblastic cells [38]. However, other cells including pericytes have also been previously shown to be involved in the vascular calcification process [8], [9] and their potential as mesenchymal precursors has recently been highlighted [39]. Our immuno-histological results showed no difference in terms of smooth muscle cells and endothelial cells presence between symptomatic and asymptomatic plaques. In contrast, higher pericyte cell density was noted in asymptomatic lesions, suggesting that pericytes could be actively involved in plaque stability.

The most studied potential sources of OPG in the artery wall are endothelial cells and smooth muscle cells. Upon inflammatory stimulation, these cells release OPG in the surrounding extra-cellular matrix as well as in the circulation, which is one of the possible sources of circulating OPG in atherosclerotic patients [40]. Based on *in vitro* preliminary results, we observed that unstimulated pericytes had an intense secretion of OPG at baseline in comparison to smooth muscle cells and endothelial cells (Figure S3 in File S1). Altogether, these notions led us to hypothesize that vascular pericytes could play a critical role in arterial calcification formation and regulation, similar to that of osteoblasts in bone tissue. To confirm this hypothesis, we first assessed the presence of pericytes in our cohort of carotid plaques. We observed CD146^+^ and NG2^+^ pericyte cell presence in the lesions but with a significantly more intense staining in the asymptomatic carotid plaques. Of note, no significant difference was noted in terms of CD31^+^ endothelial staining nor in terms of actin staining, further suggesting that pericytes could be responsible for this difference (Fig. 2). Then, we assessed the osteoblastic potential of human primary pericytes *in vitro.* We showed that human pericytes were able to differentiate into osteoblast-like cells and to mineralize (Fig. 4A and 4B). Finally, we found that pericytes secreted increasing amounts of OPG during osteoblastic differentiation and upon inflammatory stimulation by IL-6 and TNF-α (Fig. 4D). In other words, pericytes, that have the ability to differentiate into osteoblasts and to secrete high amounts of OPG were found in a significantly higher proportion in the asymptomatic plaques that were precisely characterized by an intense presence of both pericyte cells, OPG, as well as a higher proportion of osteoid metaplasia.

As in bone tissue, it is hypothesized that in ectopic calcification, mineral deposition and resorption are two opposite processes tightly balanced and regulated by osteoblast- and osteoclast-like cells. Cells from the monocytic lineage are most likely the precursors of the giant multinucleated cells observed in atherosclerosis lesions [10]. Following on with this hypothesis, we observed that pericytes supernatant inhibited the differentiation of Hu- CD14^+^ cells into osteoclasts, an effect that persisted under IL-6 and TNF-α inflammatory stimulation (Fig. 4C). Given the intense secretion of OPG by pericytes during osteoblastic differentiation and under inflammatory stimulation (Fig. 4D), we hypothesized that this inhibitory effect was OPG-mediated. Indeed, blockage of OPG using a neutralizing antibody proved efficient in significantly reversing this inhibition (Fig. 4E).

Altogether, these results suggest that pericytes are implicated in the formation of vascular calcification. Resident vascular pericytes may have a protective effect against the development of vascular calcification by regulating the balance of mineral formation together with other cells such as monocytes/macrophages. Exposure to inflammatory atherosclerotic stress induces pericytes to (i) differentiate towards an osteoblastic lineage and (ii) secrete various mediators, including OPG, which trigger an imbalance between mineral formation and resorption in the plaque. As a result, intense calcification, and in some cases OM, develop within atherosclerotic lesions which ultimately may stabilize the plaque.

### Study limitations and perpesctives

Our results suggest that OPG is an interesting predicitive marker for carotid plaque vulnerability in patients presenting with carotid artery stenosis, however the plasma evaluation is based on a the analysis of a single blood sample harvested the day before surgery. A clinical study comprising serial plasmatic OPG evaluations of symptomatic and asymptomatic patients is warranted to confirm the relevance of OPG as a predictive factor for carotid plaque stability in routine clinical practice.

The intense expression of OPG in asymptomatic carotid lesions is associated with the presence of OM. Whether OPG is secreted in response to atherosclerosis-related stress and fosters the development of bone-like vascular calcification or is secreted as a retaliatory metabolite that is finally overwhelmed is not clear and further studies are needed to address this aspect.

Finally, the presence of OM seems to be noted consistently and exclusively in the asymptomatic lesions, making OM an interesting qualitative marker for plaque composition. However, to our knowledge, no imaging modality can distinguish OM from other types of vascular calcification.

## Conclusion

Our study shows that asymptomatic and symptomatic carotid lesions are completely opposed in terms of OPG and calcic patterns. Asymptomatic plaques are characterized by an intense mineralization with often the presence of OM, a high OPG and pericytes presence as well as elevated circulating OPG levels. These completely different profiles suggest that circulating OPG could be a useful plasmatic predictive factor for plaque vulnerability in clinical practice. Furthermore, our studies indicate that OPG and pericytes are governing factors in the develoment of arterial calcification.

## Supporting Information

File S1 **File S1 contains Table S1 and Figures S1, S2 and S3.**
(DOCX)
