# Supplementary material for: Osteoprotegerin, Pericytes and Bone-Like Vascular Calcification Are Associated with Carotid Plaque Stability
Source: PLoS One. 2014 Sep 26;9(9):e107642. doi: 10.1371/journal.pone.0107642 (PMC4178031; doi:10.1371/journal.pone.0107642)
Supplement: File S1 — contains Table S1 and Figures S1, S2 and S3. (DOCX) [file pone.0107642.s001.docx]

**Supplementary Tables**

**Table S1:** Demographic parameters, cardiovascular risk factors, cardiovascular co-morbidities and treatment of patients operated on for either symptomatic (SC) or asymptomatic (AsC) carotid artery stenosis.

|  | **Carotids** | | | **p-value SC vs AsC** |
| --- | --- | --- | --- | --- |
|  | **SC** | **AsC** | **Total** |  |
| **Age** |  |  |  | 0.490 |
| **n** | 24 | 49 | 73 |  |
| **mean±SD** | 71±9 | 69±11 | 70±11 |  |
| **BMI** |  |  |  | 0.070 |
| **n** | 24 | 48 | 72 |  |
| **mean±SD** | 24.6±3.6 | 26.6±4.7 | 25.9±4.4 |  |
| **LDL (g/L)** |  |  |  | **0.020** |
| **n** | 23 | 44 | 67 |  |
| **mean±SD** | 0.7±0.3 | 0.9±0.3 | 0.9±0.3 |  |
| **HDL (g/L)** |  |  |  | 0.270 |
| **n** | 23 | 44 | 67 |  |
| **mean±SD** | 0.5±0.2 | 0.6±0.2 | 0.5±0.2 |  |
| **Female, n(%)** | 6 (25%) | 14 (29%) | 20 (27%) | 0.75 |
| **Smoking, n(%)** | 10 (42%) | 31 (63%) | 41 (56%) | 0.08 |
| **HTA, n(%)** | 18 (75%) | 45 (92%) | 63 (86%) | 0.06 |
| **Diabetes mellitus, n(%)** | 2 (8%) | 11 (22%) | 13 (18%) | 0.12 |
| **CAD, n(%)** | 12 (50%) | 27 (55%) | 39 (53%) | 0.68 |
| **PAD, n(%)** | 4 (17%) | 19 (39%) | 23 (32%) | **0.05** |
| **CVD, n(%)** | 24 (100%) | 7(14%) | 30 (41%) | <0.001 |
| **CEI, n(%)** | 6 (25%) | 24 (49%) | 30 (41%) | 0.07 |
| **Sartan, n(%)** | 7(29%) | 13 (27%) | 20 (27%) | 0.81 |
| **Statin, n(%)** | 21 (88%) | 39 (80%) | 60 (82%) | 0.32 |
| **Fibrates,** **n(%)** | 1 (4%) | 0 (0%) | 1 (1%) | 0.1 |
| **APA, n(%)** | 23 (96%) | 48 (98%) | 71 (97%) | 0.55 |
| **Aspirin, n(%)** | 16 (67%) | 33 (67%) | 49 (67%) | 0.95 |
| **Clopidogrel, n(%)** | 19 (79%) | 27 (55%) | 46 (63%) | **0.04** |
| **2 APA, n(%)** | 12 (50%) | 13 (27%) | 25 (34%) | **0.05** |
| **VKA, n(%)** | 0 (0%) | 1 (2%) | 1 (1%) | **<0.0001** |

**Supplementary Figures**

**Figure S1:**


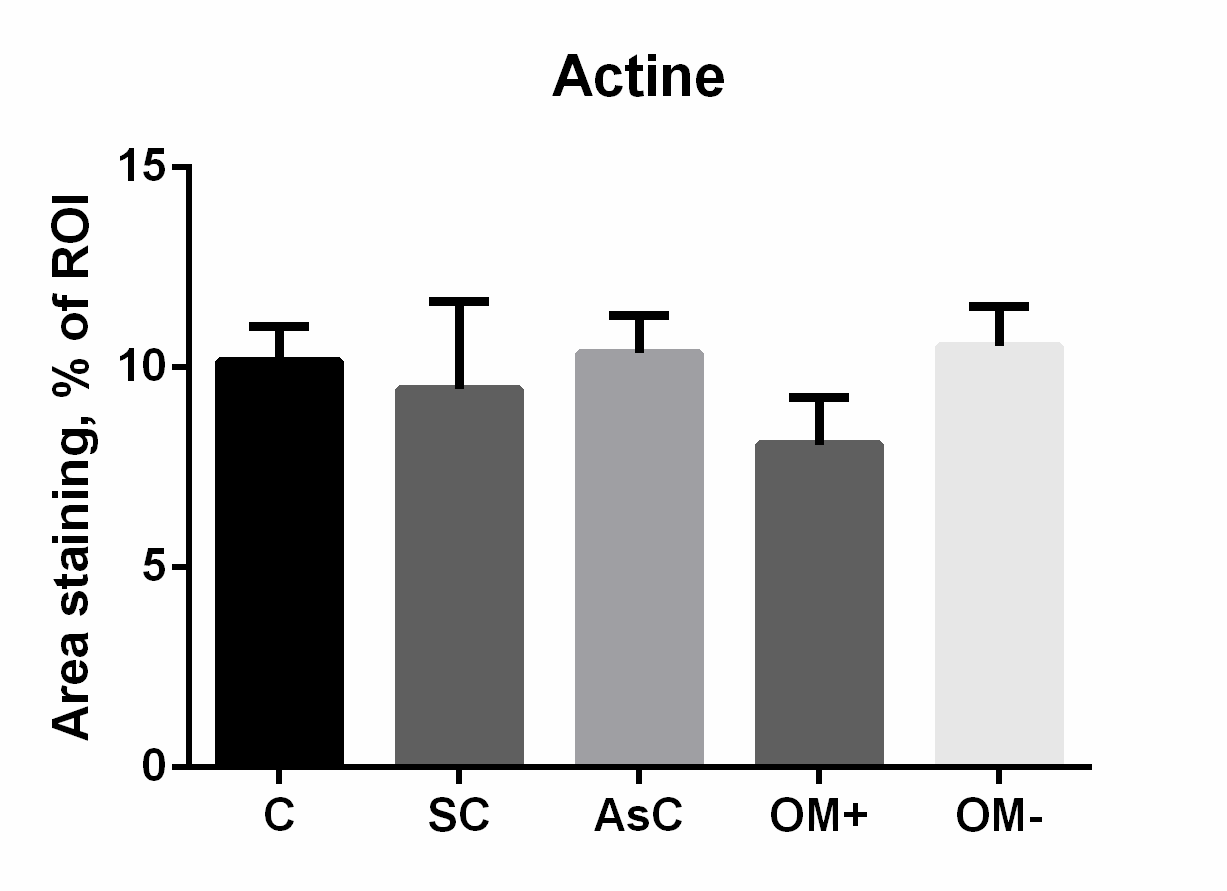


**Figure S2 :**

**Figure S3:**





***

**Supporting Information Legends**

**Figure S1:** Smooth muscle actin antibody staining showed no difference between the various groups of carotid (C), symptomatic (SC) and asymptomatic (AsC) carotid plaques and also between OM+ (presence of OM), OM- (absence of OM) lesions.

**Figure S2 :** Human primary pericytes were characterized using flow cytometry analysis as CD31-, CD146+ and NG2+ cells, MFI : Mean Fluorescence Intensity.

**Figure S3:** Elisa experiments were performed on different cell types: human primary pericytes (Pericytes), smooth muscle cells and endothelial cells harvested from carotid plaques (SMC plaque and EC plaque, respectively), and smooth muscle cells harvested from cadaveric donors before multi-organ transplantation procedure (SMC). Cells were plated at the same density (70000cells/ well) in 6 well plates. After reaching confluence, cells were cultured for 48h with 1% FCS, after which the supernatants were centrifuged, aliquoted and stored at -20°C. ***: *P*< 0.001
